# The Vascularized Fibula as Salvage Procedure in Extremity Reconstruction: A Retrospective Analysis of Time to Heal and Possible Confounders

**DOI:** 10.3390/life14030318

**Published:** 2024-02-28

**Authors:** Christian Smolle, Judith C. J. Holzer-Geissler, Patrick Mandal, Jessica Schwaller, Gert Petje, Johannes Rois, Lars-Peter Kamolz, Werner Girsch

**Affiliations:** 1 Division of Plastic, Aesthetic and Reconstructive Surgery, Department of Surgery, Medical University of Graz, 8036 Graz, Austria; 2 Orthopaedic Hospital Speising, 1130 Vienna, Austria; 3 AUVA Trauma Centre Meidling, 1120 Vienna, Austria

**Keywords:** extremity reconstruction, vascularized fibula graft, bone graft, osseous incorporation, healing time, gender disparities, microsurgery, outcome

## Abstract

The vascularized fibula transfer is a well-established technique for extremity reconstruction, but operative planning and patient selection remains crucial. Although recently developed techniques for bone reconstruction, such as bone segment transfer, are becoming increasingly popular, bone defects may still require vascularized bone grafts under certain circumstances. In this study, 41 cases, 28 (68%) men and 13 (32%) women (median age: 40 years), were retrospectively analyzed. Therapy-specific data (flap vascularity [free vs. pedicled] size in cm and configuration [single- vs. double-barrel], mode of fixation [internal/external]) and potential risk factors were ascertained. Indications for reconstruction were osteomyelitis at host site (*n* = 23, 55%), pseudarthrosis (*n* = 8, 20%), congenital deformity (*n* = 6, 15%), traumatic defect, and giant cell tumor of the bone (*n* = 2, 5% each). Complete healing occurred in 34 (83%) patients after a median time of 6 months. Confounders for prolonged healing were female gender (*p* = 0.002), reconstruction in the lower limb (*p* = 0.011), smoking (*p* = 0.049), and the use of an external fixator (*p* = 0.009). Six (15%) patients required secondary limb amputation due to reconstruction failure, and one patient had persistent pseudarthrosis at last follow-up. The only risk factor for amputation assessed via logistic regression analysis was preexisting PAOD (peripheral artery occlusive disease; *p* = 0.008) The free fibula is a reliable tool for extremity reconstruction in various cases, but time to full osseous integration may exceed six months. Patients should be encouraged to cease smoking as it is a modifiable risk factor.

## 1. Introduction

Limb salvage is one of the key hurdles of reconstructive surgery. Salvage operations due to osteomyelitis, trauma, and cancer do often result in large bone defects. While there are options of non-vascularized bone grafts, these have a high rate of non-incorporations and hence lead to non-union of the bone or to pseudoarthrosis. In addition, high rates of infections and unusual fractures have been reported [1,2,3,4,5,6]. For these reasons, the vascularized fibula graft is a commonly applied procedure for the reconstruction of long-bone defects. The fibula, being an expandable bone bearing only 17% of the body weight, has been shown to be a reliable vascularized graft with a very consistent vascular pedicle, i.e., the peroneal vessels [7,8]. In its vascularized form, the fibula graft has a high rate of incorporation and thus provides the possibility to reconstruct otherwise compromised extremities [7]. Though being a comparably small-caliber bone, it usually undergoes hypertrophy more than two times its original size to match the diameters of the reconstructed bone over time—a property that cannot be observed in non-vascularized bone grafts [9]. On a cellular level, these conventional bone grafts undergo a phase of osteocyte necrosis, intramedullary fibrosis, and a subsequent process that has been coined “creeping incorporation”; i.e., an ingrowth of adjacent vessels and the replacement of necrotic bone. Conversely, the viability of osteocytes in vascularized grafts has been seen to exceed 50%, thus preventing extensive tissue necrosis and reducing the need for the substitution of the entire bone matrix over time [10]. Furthermore, the vascularized bone provides an ostoeinductive signal and has the advantage of being relatively impervious against the vascular impairment of the surrounding tissues [11]. Besides the reconstruction of bone defects, the vascularized fibula provides options to be used not only as an osseous flap but also the possibility to be transferred as an osteocutaneous or osteomyocutaneous flap for the potential coverage of additional soft tissue defects. Further modifications include its transfer with its proximal joint surface and growth plate, or inset in a folded, so-called “double-barrel” fashion, to reconstruct combined defects of one or both forearm bones with one graft or to bridge defects in larger bones such as the tibia or femur [12]. A further method of application in large bones that has been proposed by Capanna uses the fibula in combination with an allograft that serves as a scaffold for subsequent bone regeneration that is enhanced by the adjacent vascularized bone [13,14]. A recent meta-analysis found higher healing rates in vascularized bone grafts when compared to non-vascularized grafts, albeit there was no significant difference with regard to complication rates [15].

Masquelet’s induced membrane technique was first performed by the namesake in the mid-1980s and is a two-stage procedure in which the osteomyelitic bone segment is resected and a polymethyl methacrylate bone cement spacer is inserted into the defect as a first step. After the formation of an autologous and presumably well-vascularized foreign body membrane around the spacer, a non-vascularized bone matrix is inserted into the defect. In theory, the bone is nourished via this membrane; however, the precise mechanism has not yet been fully understood [16]. When treating osteomyelitic bone, vascularized fibula transfer has advantages over the conventional Masquelet’s technique or bone segment transports. With this technique, the defect may be reconstructed immediately with well-vascularized tissue, and secondary procedures are usually only necessary when fixation devices have to be removed. Furthermore, healing of a vascularized bone graft usually occurs within 6 to 9 months, whereas (depending on the distance of the bone defect), both Masquelet’s and bone segment transport techniques have the disadvantages of at least two surgical procedures being needed (Masquelet’s) and longer healing times of 10 (Masquelet’s) to 17 (bone segment transfer) months [17]. Despite being a versatile flap for extremity salvage, the most common complications include infection, soft-tissue necrosis (when incorporated), and non-union with the need of reoperation.

With this study, we aimed to assess healing times and potential confounders, as well as complications and possible risk factors, after vascularized fibula transfer for extremity reconstruction. We also intended to demonstrate that vascularized fibula transfer is a feasible reconstructive procedure irrespective of patient age or the origin of the bone defect that still has its indications in patients where other reconstructive measures have failed or have become unfeasible.

## 2. Patients and Methods

### 2.1. Study Design

**Figure 1:**
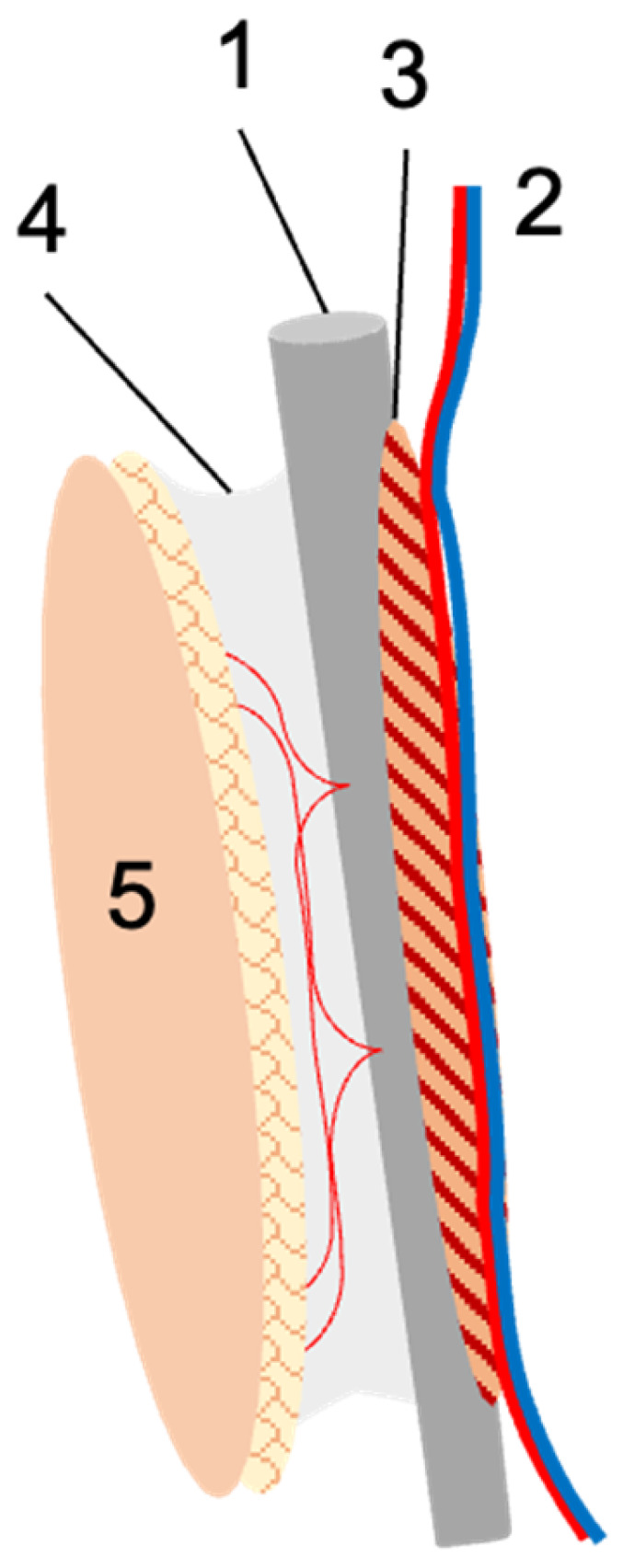
Schematic illustration of the fibula flap. The fibula (1) is harvested with the nourishing peroneal vessels (2), and for optimal periosteal perfusion, usually a small muscular cuff (3) is included. If needed, the lateral intermuscular septum with its perforating vessels (4) is harvested with the flap, upon which a skin island (5) can be based. In the case of free fibula transfer, the peroneal vessels are re-anastomosed at the recipient site. In the case of pedicled transfer, the fibula remains in the donor leg and is used for tibial reconstruction. The vascular pedicle is transected either distally or proximally to obtain a proximally or distally pedicled fibula graft.

Patients that had received a vascularized fibula graft as a salvage procedure for extremity reconstruction between 1 January 2000 and 31 December 2013 were retrospectively analyzed. All patients had been operated on either at the Orthopedic Hospital Speising, Vienna, Austria, or the Trauma Hospital Meidling, Vienna, Austria, by the same plastic surgeon. Therefore, this study can be considered monocentric. Inclusion age ranged from 2 to 70 years of age. Patients were only excluded if no postoperative follow-up data were available. All data were ascertained from patient records and encompassed the following patient-specific data: age at the time of reconstruction, gender, affected limb, affected bone, indication for the procedure, whether or not surgical pretreatment had been carried out, comorbidities, and last follow up. Therapy-specific data included fibula flap vascularity (free, proximally, or distally pedicled), osseous configuration (single- or double-barrel), flap design (osseous, osteocutaneous, osteomyocutaneous), mode of osseous fixation (internal vs. external), and surgery time. Outcome measures included time to full osseous integration (i.e., proximally and distally), as confirmed by plain radiographs taken at least two, three, and six months postoperatively (and thereafter at least until complete healing); complications; and the need for secondary amputation. Figure 1 schematically illustrates the fibula flap with the relevant anatomy.

### 2.2. Patient Preparation and Surgical Technique

After an indication for reconstruction with a vascularized fibula graft had been established, all patients received vascular imaging (MRI or CT angiography) to check for relevant vascular anomalies or stenoses, which were salvaged at least one month prior to surgery whenever present. All surgeries were carried out under general anesthesia. Induction was carried out using 2 mg/kg Propofol (4–6 mg/kg in children), 0.6 mg/kg Rocuronium, and 0.5 µg/kg Remifentanil. Anesthesia maintenance was achieved using Sevoflurane with a minimal alveolar concentration of 0.7. Analgesia was achieved with the repeated administration of Remifentanil throughout the procedure, and upon surgical requirement, Rocuronium was given. In patients with contraindications for Sevoflurane, total intravenous anesthesia was carried out using Propofol and Remifentanil with Rocuronium as needed. In all cases, the fibula was harvested together with the peroneal vessels and a small muscular cuff for optimal blood supply. If needed for soft tissue coverage, a skin island based on the septocutaneous perforators was harvested with the fibula. The fibulae were transferred either proximally or distally pedicled or as free flaps. Osseous fixation was accomplished dependent on the local situation and individual requirements by means of plates, screws, nails, or external Ilizarov fixators.

### 2.3. Statistics

Statistical analysis was performed with SPSS 29.0 (IBM Inc., Armonk, NY, USA). Univariate analysis was performed using the Chi^2^ test for dichotomous variables, and Spearman’s correlation analysis was carried out for ordinal variables. To test for the normal distribution of continuous parameters, Levene’s test was applied. Student’s *t*-test was used for normally distributed variables; otherwise, the Mann–Whitney-U test was used. Kaplan–Meier charts were created to assess for duration to full osseous integration and, Cox regression was applied to identify influential factors for it. Binary stepwise backwards logistic regression analysis was applied to assess the influence of multiple confounders on dichotomous variables. In all cases, a *p*-value below 0.05 was considered as statistically significant. For a more detailed description of the statistical procedures, see Brosius (1998) [18].

## 3. Results

### 3.1. Patients and Indications

For detailed patient data, see Table 1. Thirty-nine patients were included in the study, of which two received two fibula grafts at different time points and hence were treated as two individual cases each. This resulted in a total number of 41 transferred vascularized fibulae. Twenty-eight (68%) fibulae grafts were performed in men, and thirteen (32%) were performed in women. The median age at the time of surgery was 40 years (interquartile range [IQR] 18–47.5), with a range of 2 to 70 years.

In 34 cases (83%), the lower limb was affected, and the tibia was the most commonly affected bone (*n* = 26, 63%, see Table 2). Indications for reconstruction were osteomyelitis (*n* = 23, 56%), pseudarthrosis (*n* = 8, 20%), congenital deformity (*n* = 6, 15%), post-traumatic osseous defect, and giant cell tumor of the bone (*n* = 2, 5% each). In 21 patients (52%), there was an additional soft tissue deficiency, and in 13 (32%), an adjacent joint was affected by the bone defect. The vast majority (*n* = 34, 83%) had received surgical pretreatment, of which 27 (66%) had had more than one prior surgical intervention.

### 3.2. Potential Risk Factors

Twenty-three (56%) had at least one potential risk factor, encompassing peripheral artery occlusive disease (PAOD; *n* = 5, 12%), arterial hypertension (*n* = 4, 10%), osteoporosis (*n* = 4, 10%), obesity (*n* = 2, 5%), alcoholism, and diabetes mellitus and malnutrition (*n* = 1, 2% each). Nineteen (46%) patients were active smokers at the time of surgery. There was a strong correlation between higher age and the presence of potential risk factors (*p* < 0.001). Smoking was significantly more common in men (57 vs. 23%, *p* = 0.042). Otherwise, no gender-related differences could be detected.

### 3.3. Surgical Procedure

In the majority of cases (*n* = 23, 56%), the fibula was transferred as free flap, and most fibulae were inserted in a single-barrel fashion (*n* = 28, 68%), of which three (7%) were transferred along with the fibula head for joint reconstruction. External fixation methods were more often used than internal fixation methods (*n* = 25, 61% vs. *n* = 16, 39%, respectively), and the most common flap design was osteocutaneous (*n* = 25, 61%). The median bone graft length was 13 cm (IQR 8–22.5), and the median surgery time was 7.5 h (IQR 6.4–10; for detailed data, see Table 3).

### 3.4. Postoperative Complications, Revision Surgeries, and Secondary Amputation Rates

Postoperative complications requiring revision surgeries occurred in 11 (27%) patients. In six (15%) patients, secondary limb amputation was necessary due to recurrent osteomyelitis (*n* = 3, 7%), graft failure (*n* = 2, 5%), and acute extremity ischemia after surgery (*n* = 1, 2.5%). Amputation was only performed in men and in the lower limbs after a median time of 7 months (IQR: 4.63–29.25, see also Figure 1). Further complications encompassed pseudarthrosis formation (*n* = 3, 7%), external fixator pin loosening (*n* = 1, 2.5%), and fracture of the host bone (tibia, *n* = 1, 2.5%). This necessitated revision surgery in all of these cases after a mean time of 10 months (IQR: 6.5–19.5), resulting in eventual healing in four of five patients. One patient (2.5%) had persistent pseudarthrosis at last follow up and hence had to be excluded from our healing time analysis.

### 3.5. Time to Heal

**Figure 2:**
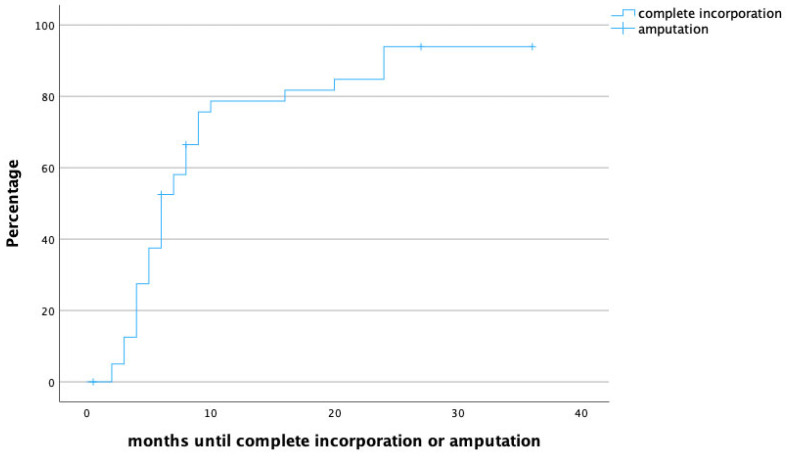
Time to heal in months. Censored cases are patients that required secondary amputation of the limb.

The median follow-up time was 20 months (IQR 8.5–31.5), and the median time to full osseous integration was 6 months (IQR 4–8.25) (Figure 1 demonstrates time to heal by means of a survival chart). The median time to proximal incorporation was 6 months (IQR 4–8), slightly longer than time to distal incorporation (median 5, IQR 4–8 months). Figure 2 displays time to heal by means of an inverted survival chart. The higher the curve, the more fibulae have successfully healed.

### 3.6. Confounders for Healing Time

**Figure 3:**
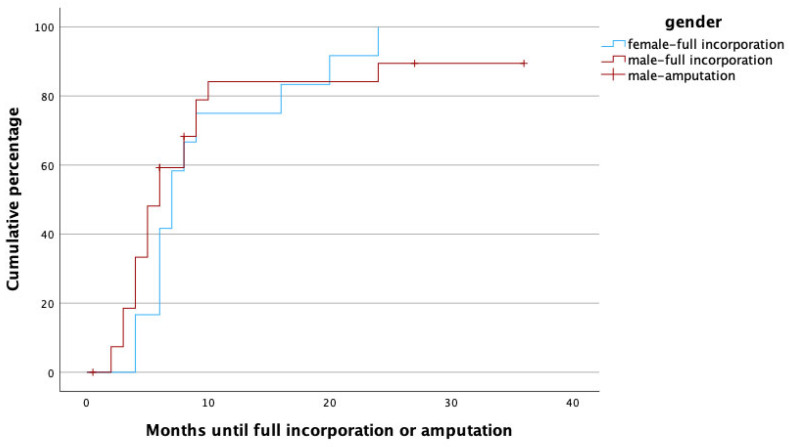
Time to heal in months for male (red) and female (blue) gender. Note that amputations (censored cases) were only performed in male patients.

**Figure 4:**
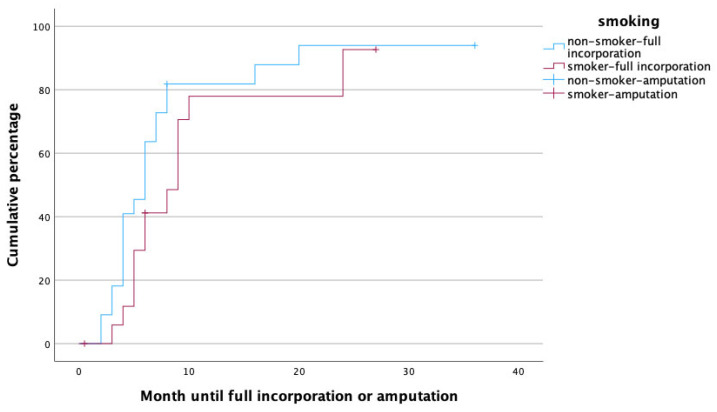
Time to heal in non-smokers (blue) and smokers (red).

**Figure 5:**
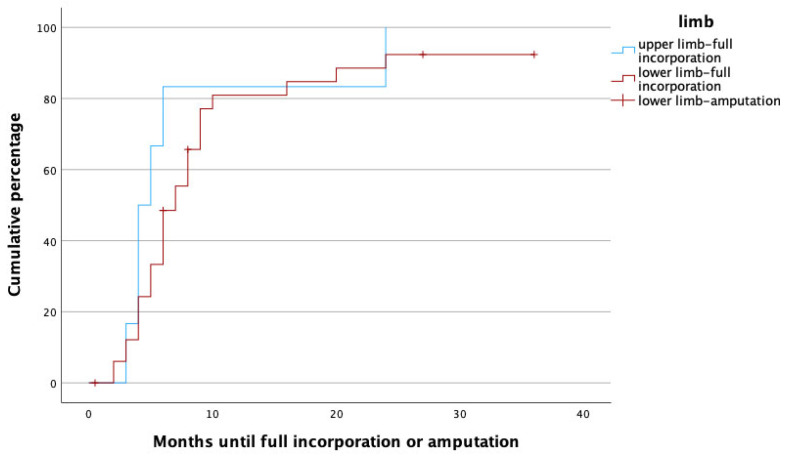
Time to heal in upper (blue) and lower (red) limbs. Note that amputations (censored cases) only had to be performed in lower limbs.

**Figure 6:**
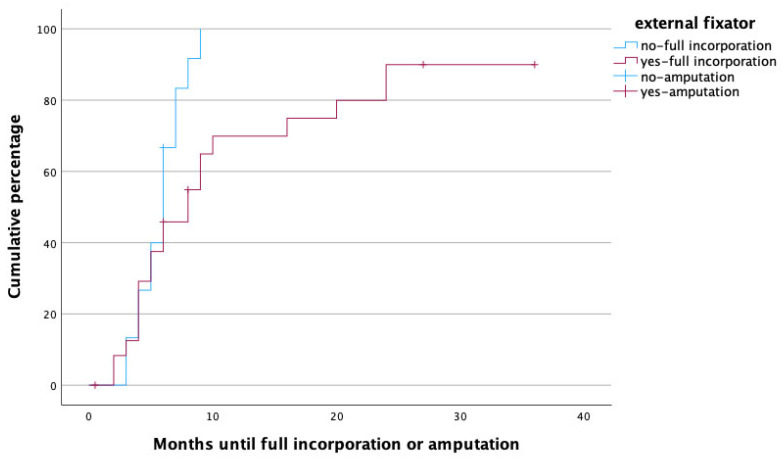
Time to heal in months for patients treated with (red) and without (blue) external fixator. Note that amputations (censored cases) were more frequently performed in patients with external fixator.

To identify potential risk factors for complete graft incorporation, a Cox regression analysis was performed, with the potential confounders being set as age, gender, affected limb (upper vs. lower) risk factors (PAOD, arterial hypertension, osteoporosis, obesity, alcoholism, diabetes mellitus, malnutrition, osteomyelitis as treatment indication, active smoker), surgery time, fibula configuration (single- vs. double-barrel), fibula length and vascularity (free vs. pedicled), and method of bone fixation (internal vs. external fixation) in relation to healing time. With this analysis, female gender (*p* = 0.002, HR: 0.172, 95% CI [0.056:0.526]), smoking (*p* = 0.049, HR: 0.208, 95% CI [0.044:0.992]), the use of an external fixator (*p* = 0.009, HR: 0.168, 95% CI [0.044:0.640]), and reconstruction in the lower limb (*p* = 0.011, HR: 0.183, 95% CI [0.049:0.680]) were significant predictors for prolonged healing. Figure 3, Figure 4, Figure 5 and Figure 6 illustrate healing times for both genders, smokers and non-smokers, patients with and without an external fixator, and reconstruction of the upper and lower limb, respectively, and they should be interpreted in the same fashion as Figure 2.

### 3.7. Patients with Secondary Amputations and Risk Factor Analysis

As mentioned above, secondary lower limb amputation became necessary in six male patients with an age ranging from 37 to 52 years. Reconstruction was performed for pseudarthrosis in one patient and for osteomyelitis in five patients. In five patients, the tibia was reconstructed, and in one patient, the calcaneus was reconstructed. Two fibulae were transferred as free, two as distally pedicled flaps, and two as proximally pedicled flaps.

In the binary stepwise forwards logistic regression analysis using the same variables as above, the presence of PAOD (*p* = 0.008) was the sole predictor for secondary amputation. Although amputations only had to be performed in men and the lower limbs; neither were significant confounders for therapy failure when tested independently using the Chi^2^ test (*p* = 0.071 and 0.229, respectively).

### 3.8. Donor Site Morbidity

Long-term donor site morbidity was seen in three patients in total and encompassed persistent partial peroneal nerve paralysis in two (5%) cases and upper ankle joint instability in one (2%) case.

## 4. Discussion

In this retrospective study, the reconstructive outcome of vascularized fibula grafts used for extremity reconstruction was reviewed. Although the overall secondary amputation rate was low (15%), healing time of the bone graft exceeded six months in almost half of the patients. Female gender and the use of an external fixator was associated with prolonged healing times. To the best of our knowledge, this is the first study exclusively evaluating factors with a potential impact on the osseous incorporation of free fibula grafts in all age groups—children, adolescents, adults, and the elderly.

The median healing time of the vascularized fibula grafts was six months in our cohort, which is slightly longer than in comparable cohorts described by Xu et al., who found a mean healing time of 4.9 months [19], and Lefebvre et al., who observed a mean time to union of 19.9 weeks or 4.6 months [20] of vascularized fibula grafts. The comparably longer healing time in our patients might also have been due to the usual follow up regimen that included plain radiographs at discharge, as well as at one, two, three, and six months after surgery. For this, earlier full osseous incorporation might be adequately reflected by the present data.

Patients with various indications for reconstruction were included in the study. Chronic osteomyelitis was by far the most common treatment indication. However, no correlation between healing time or amputation rate and former host site osteomyelitis was observed. This observation aligns well with those of previous studies on extremity reconstruction with vascularized bone grafts. The patent muscular and osseous vascularity obviously permits their use in infected surroundings and ensures event-free healing, provided meticulous debridement has been performed beforehand [21]. Furthermore, it is advised to leave a muscular cuff attached to the bone graft to enhance the delivery of antibiotics and immune components to the recipient site [13,22], and this was carried out in all of our patients. In the presented cohort, recurrence of osteomyelitis was seen in 3 of 23 cases (13%). This is lower than in the cohort described by Ciclamini et al., who found recurrent fistulae in 3 of 14 (21%) osteomyelitis cases treated with vascularized bone flaps in single-stage procedures [23]. In all of our patients with recurrent osteomyelitis, this complication ultimately necessitated secondary limb amputation.

Female gender was independently associated with longer healing times when compared to male gender. In fact, full osseous incorporation occurred five times more rapidly in male patients. Guidi et al. compared the healing rates of vascularized and non-vascularized bone grafts in the treatment of long bone non-union and found that male gender was related to a 48% shorter healing time [24]. Although there is evidence concerning gender-related differences with regard to bone healing, the medical literature is still inconclusive concerning the extent of the effects. On a molecular level, estrogen in high doses has been seen to reduce the serum availability of insulin-like growth factor 1 (IGF-1), a growth hormone with high relevance to bone healing, whereas testosterone seems to increase IGF-1 levels. Furthermore, men tend to have higher bone density than women [25]. In a spinal fusion model in rats, higher fusion rates were observed in male individuals [26]. Clinical observations have yielded somewhat contradictory results: Chang et al. found significantly higher non-union rates in females following scaphoid reconstruction with vascularized bone grafts [27], whereas a meta-analysis analyzing tibial fracture non-unions found that male gender was a risk factor for pseudarthrosis [28]. Some clinical studies could not detect any differences in osseous healing time at all [29,30]. Animal studies in rats suggested that compromised bone healing in females might also be related to decreased mesenchymal stem cell activity [31]; this finding has thus far, however, not been reproduced in human subjects. Obviously, the reasons for possible disparities in bone healing seem to be multifactorial and reach from molecular factors to differences in bone structure and, last but not least, different lifestyles among women and men.

As one may have expected, active smoking was related to prolonged time to full incorporation. The adverse effects of smoking on fracture healing have been reported before on various occasions. For instance, smoking has been seen to prolong the healing times of tibial fractures or spinal fusions, as well as elective knee and foot surgery [30,32,33,34]. The same goes for conventional and vascularized bone grafts in upper and lower extremity reconstruction, where smoking was associated with a 75% decreased healing rate, and the number of pack-years correlated with longer healing times [24]. In fact, it has even been demonstrated that the amount of nicotine intake is closely correlated with healing times [34]. Conversely, smoking cessation after bone surgery for six months has been seen to reduce healing times so that it almost reaches non-smoker levels [33]. In our cohort, neither the amount cigarettes smoked nor the rate of smoking cessation after surgery were assessed. However, being an independent predictor of prolonged healing in our cohort, even stronger than PAOD (which was associated with the need for secondary amputation), age or gender, its adverse effects on unsuccessful healing of the vascularized fibula is obvious.

It is well known that the process of fracture healing takes longer in the more weight-bearing bones of the lower limbs. Also in our patients, expectedly, the incorporation of the vascularized fibula took significantly longer in the lower limbs. Xu et al. reviewed 18 patients who received fibula grafts for extremity reconstruction after osteosarcoma resection and found slightly longer healing times (5.1 vs. 4.8 months) in the lower limbs. Interestingly, the difference was so small that it did not reach statistical significance in that study [19]. Similar observations were presented by Guidi et al., who reviewed the healing times of vascularized and non-vascularized bone grafts and found higher healing rates and quicker incorporation in the upper limbs as well [24].

The use of an external fixator for bone stabilization as opposed to internal fixation with plates was an independent predictor for prolonged fracture healing. Similar observations have been described by Berven et al., who performed a case–control study comparing tibial fractures managed with Ilizarov frames or locking plates; despite the fact that injury severity was higher in the locking plate group, fracture healing took significantly longer in the Ilizarov group. These results are further supported by the fact that selection bias was ruled out as patients from two different centers, one using mainly locking plates and one using mainly Ilizarov frames, were compared. The mode of fixation, however, had no influence on the rates of pseudarthrosis formation or deep infections [35]. This is in line with our findings, as the mode of fixation obviously had no influence on therapy failure and subsequent amputation rate in our cohort. It has to be pointed out that the mode of bone fixation was, in each individual case, a carefully taken decision based on the requirement of stability of the limb rather than a matter of choice or surgeon’s preference. Therefore, an adequate comparison between the two modes might not be possible based on the retrospective data available to us. It is possible though that external fixation was deemed suitable especially for the more complex reconstructive procedures, and the data have been biased in this respect.

Secondary limb amputation became necessary in six patients and only in men and in the lower limbs. Both of these parameters were not associated with the risk for secondary amputation. Although fibula transfer was only performed in patients without relevant stenosis of lower extremity vessels at the time of surgery (i.e., vascular stenoses had been salvaged before the procedure), PAOD was the sole significant predictor for secondary limb amputation. In the medical literature, however, not even severe PAOD with only one patent lower leg vessel is regarded as an absolute contraindication for the microvascular reconstruction of the lower limb. What is more, it has been demonstrated that even complicated and chronic tissue defects can be salvaged under such unfavorable conditions [36]. Although complication rates after free flap procedures in the lower limbs are higher in patients with PAOD, this does not necessarily affect the long-term reconstructive outcome, as demonstrated in a study by Bovill et al., who observed comparable success rates in patients with and without PAOD [37]. While steal phenomena after microvascular reconstruction in the lower limbs represent a well-known and feared complication [38], postoperative ischemia of the operated leg was the immediate reason for amputation in just one of five patients. In two cases, the need for amputation arose from graft failure, and in three cases, recurrent osteomyelitis created a non-salvageable situation, and amputation was inevitable. In light of the late onset of most of the complications, creeping vascular compromise of the transplanted fibula could have been the possible reason for ultimate graft failure.

Expectedly, the rate of donor site morbidity was low. Relevant complaints arose in three patients (two with persistent peroneal nerve paralysis, one with upper ankle joint instability). Studies investigating donor site morbidity after vascularized fibula harvest have revealed that the most common issues are related to local wound complications that typically resolve over time [39]. Long-term effects are usually observed concerning gait picture (lower walking speed, decreased stride length) and sometimes stamina (decreased maximum walking distance) [39,40]. In light of the not neglectable but considerably low donor site morbidity that comes with vascularized fibula harvest, its use for extremity reconstruction in the absence of other possibilities seems justified.

The major strengths of this study are its single-center design, which ensured that all patients were treated by the same surgical standard and followed the same follow-up scheme. Furthermore, patients treated because of bone defects that arose from several indications were included in the study; this, in turn, allowed for a comparison between patients treated for septic and a-septic conditions, which, to the best of our knowledge, had not been carried out before. We were able to include patients from different age groups—from infants to the elderly—into the study; hence, we were able to demonstrate that age does not affect the outcome of the treatment.

The major limitation of this study is its retrospective character. Although, to the best of our knowledge, the present cohort represents one of the largest collectives to be analyzed in-depth, the sample size still is comparably small. Naturally, this circumstance also reflects the rare necessity for vascularized fibula transfer. The heterogeneity of the cohort can also be seen as a drawback, although neither age nor treatment indication seemed to have any impact on reconstructive outcome.

## 5. Conclusions

▪ Vascularized fibula graft is a versatile flap ideal for the reconstruction of extensive defects of long bones. Success rates are independent of patient age or treatment indication.
▪ Careful patient evaluation may be necessary in the face of PAOD, as this has been associated with therapy failure and was associated with a subsequent need for amputation in our cohort.
▪ With very limited donor site morbidity, vascularized fibula graft can be considered as an ideal method for the reconstruction of bone defects in the extremities if other treatment options have failed or cannot achieve comparably satisfactory results.
▪ If applicable, smoking cessation should be advised, as it is a modifiable risk factor and was associated with significantly prolonged healing times in our cohort.

Although methods such as bone segment transport or secondary bone distraction have recently gained increased popularity, vascularized fibula graft provides the option of bone reconstruction with a single-stage procedure. Whereas devascularized bone serves as a mere matrix for adjacent osteocytes, vascularized bone grafts provide viable bone that can be applied in complex defects of any kind.

## Figures and Tables

**Table 1:** Patient characteristics (total number *n* = 41 patients).

**Gender**	
Female	13 (32%)
Male	28 (68%)
**Affected limb**	
Upper limb	7 (17%)
Lower limb	34 (83%)
**Surgical pretreatment**	
Yes	34 (83%)
No	7 (17%)
**Risk factors**	
Obesity	2 (5%)
Alcoholism	1 (2%)
Malnutrition	1 (2%)
Osteoporosis	4 (10%)
Arterial hypertension	4 (10%)
PAOD	5 (12%)
Diabetes mellitus	1 (2%)
Smoking	19 (46%)

**Table 2:** Affected bones and indications for vascularized fibula transfer, *n* (%).

	Giant Cell Tumor of the Bone	Congenital Deformity	Pseudarthrosis	Osteomyelitis	Traumatic Defect
Upper extremity					
humerus	-	-	-	2 (5)	-
radius	2 (5)	-	-	2 (5)	-
ulna	-	-	-	1 (2)	-
Lower extremity					
femur	-	-	2 (5)	3 (7)	1 (2)
tibia	-	6 (15)	6 (15)	13 (32)	1 (2)
talus	-	-	-	1 (2)	-
calcaneus	-	-	-	1 (2)	-
Total	2 (5)	6 (15)	8 (20)	23 (55)	2 (5)

**Table 3:** Data on surgical procedure.

**Fibula blood supply**	
Free flap, *n* (%)	23 (56)
Pedicled proximally, *n* (%)	16 (39)
Pedicled distally, *n* (%)	2 (5)
**Fibula configuration**	
Single-barrel, *n* (%)	28 (68)
+ Fibula head included, n (%)	3 (7)
Double-barrel, *n* (%)	13 (32)
**Flap design**	
Osseous, *n* (%)	15 (36.5)
Osteocutaneous, *n* (%)	25 (61)
Osteomyocutaneous, *n* (%)	1 (2.5)
**Osteosynthesis**	
External fixation, *n* (%)	25 (61)
Internal fixation, *n* (%)	16 (39)
Intramedullary nail, *n* (%)	1 (2.5)
Plate, *n* (%)	14 (34)
Screws only, *n* (%)	1 (2.5)
Median defect length in cm (IQR; range)	8 (6.15–12.35; 1.5–24.6)
Median fibula length in cm (IQR; range)	13 (8–22.5; 2–30)
Median surgery time in hours (IQR; range)	7.5 (6.3–10; 2.3–18.2)

## Data Availability

Due to ethical restrictions, full data for the present study cannot be provided publicly. Data supporting the aforementioned results are available upon request from the corresponding author.
